# Primary Tuberculosis of Tonsils: Interesting Case Detected During the Histopathological Examination

**DOI:** 10.7759/cureus.59616

**Published:** 2024-05-03

**Authors:** Mohammed S Abdelwahed, Abdulhadi Samman

**Affiliations:** 1 Pathology, Faculty of Medicine, University of Jeddah, Jeddah, SAU; 2 Pathology, Faculty of medicine, Al-Azhar University, Cairo, EGY

**Keywords:** rare head and neck, ent pathology, palatine tonsil, extra-pulmonary tb, tuberculosis (tb)

## Abstract

Despite being a preventable and curable disease, tuberculosis, which mainly affects the lungs, is still a major cause of illness and death worldwide, with more than one million people dying from it each year. The affliction of the tonsils is uncommon, and isolated tonsillar tuberculosis in the absence of active pulmonary disease is an extremely rare condition that requires early and accurate diagnosis to provide proper management. Microscopic examination is one of the gold-standard tools for diagnosing tuberculosis. However, routine histopathological investigation for tonsillectomy specimens is not justified except in cases of unusual clinical or postoperative presentations. A 20-year-old female patient who experienced recurrent episodes of infections with enlarged tonsils and adenoids and showed a slightly unusual presentation was sent for a histopathology examination. Upon microscopic examination, a caseating granulomatous reaction was found, and staining for acid-fast bacilli tested positive. The patient was treated for tuberculosis of the tonsils, and their condition improved.

## Introduction

Tuberculosis is an infectious disease typically involving the lungs that is mainly caused by Mycobacterium tuberculosis and that is more often seen in developing or underdeveloped countries [1]. Two common forms of the disease are pulmonary and extrapulmonary tuberculosis. Isolated tuberculosis in the upper respiratory tract is a rare version that occurs in the absence of active pulmonary tuberculosis [1,2].

Diagnosing tuberculosis, especially extrapulmonary disease, still faces challenges in clinical practice; however, the WHO has given special attention to the essential role of early and accurate identification of M. tuberculosis [3]. A patient’s medical history and a physical examination may raise a clinician’s suspicion of tuberculosis. Laboratory testing, Ziehl-Neelsen staining, and mycobacterial culture are essential for diagnosis and must be performed in addition to histological examination for the excised lesions [4].

According to most of the previously published studies, routine histopathological examinations of adenotonsillectomy specimens in patients with no signs or symptoms of malignancy are considered dispensable and a waste of cost that increases the burden of surgery [5].

A review conducted by Moisa et al. shows that all patients who previously reported tonsillar tuberculosis were diagnosed based on histopathological examination (54.5% tonsillectomy, 45.5% by fine needle aspiration cytology) with 95.58% positive results [4].

In the Kingdom of Saudi Arabia, the prevalence of tuberculosis disease ranged from 8.5% to 23.1% in studies up to 2014. However, tonsillar tuberculosis cases are rarely reported [6,7]. This article presents a case of tonsillar tuberculosis affecting a female patient in Saudi Arabia with no symptoms of pulmonary tuberculosis that was first detected during a histopathology examination.

## Case presentation

**Figure 1:**
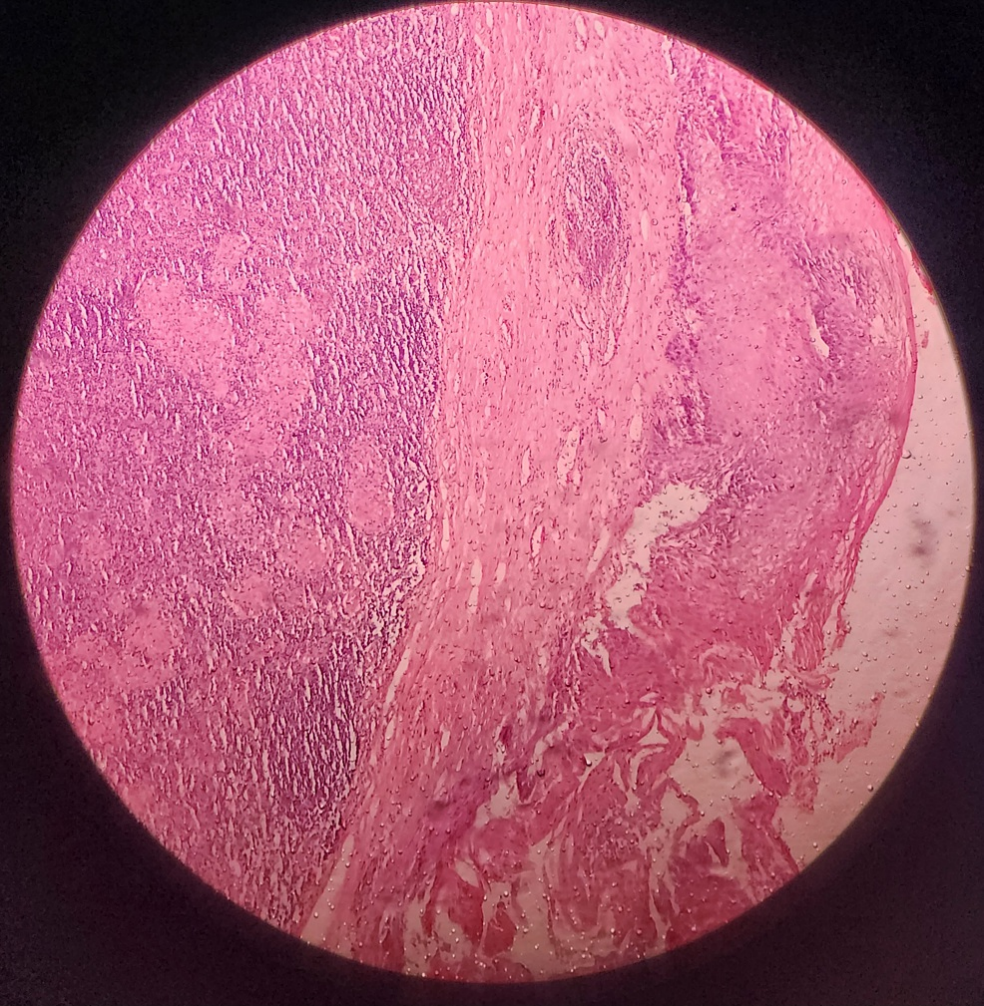
Histopathological features of tonsillar tissue showing stratified squamous epithelial capsule, lymphoid follicles, and collections of epithelioid granulomas (H&E, 100x original magnification)

**Figure 2:**
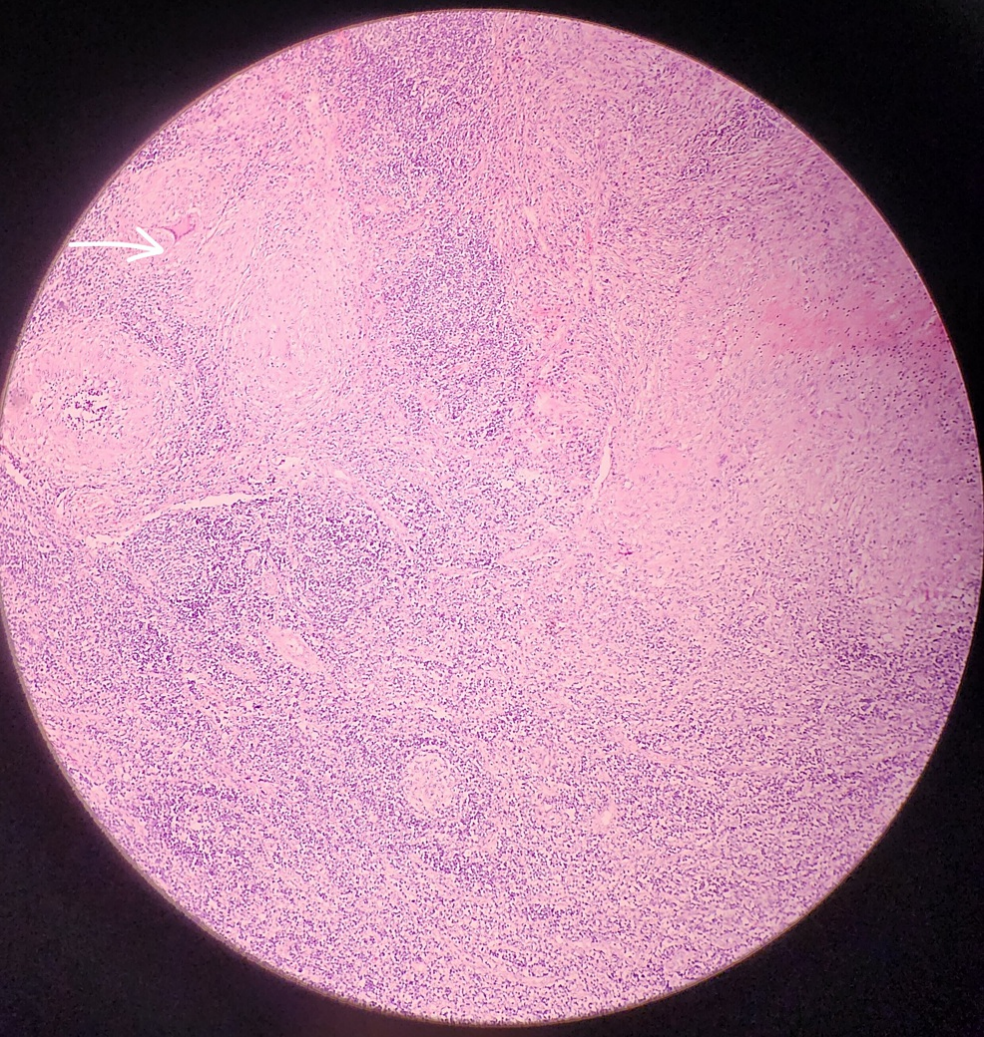
Necrotizing granulomatous reactions show lymphocytes, epithelioid cells, and Langhans multinucleated giant cells (arrow) (H&E, 200×)

A 20-year-old non-smoking woman experienced dysphagia and a loss of appetite for four months without cough, pyrosis, weight loss, or fatigue, and she had a history of snoring and mouth breathing in addition to recurrent upper respiratory tract infections, according to recorded clinical data. A physical examination of the patient revealed enlarged tonsils and adenoids that were recommended for adenotonsillectomy and histopathology. A complete blood count was requested and showed hemoglobin = 12%, white blood cells = 8.07/mm^3^ (normal range 4-11), lymphocyte count = 1.2%, and red-cell distribution width = 15.2%. Following the operation, two tonsils and adenoids specimens were received at the histopathology department in formalin and measured as follows: right tonsil 2.5 × 2 × 1.5 cm, left tonsil 2 × 2 × 1.5 cm, and adenoids 2 × 1.5 × 1.5 cm. A microscopic examination of the prepared sections from the tonsil showed multiple epithelioid granulomas within the tonsils and adenoids with Langhans-type giant cells, lymphocytes, fibroblasts, scant inflammatory infiltrate, and caseous necrosis (Figures 1, 2). 

Lymphoid follicles (with germinal centers) also showed areas of caseous necrosis that suggested tonsillar tuberculosis. Acid-fast bacilli staining on the tissue sections was positive for bacilli. The final diagnosis was rendered as tonsillar tuberculosis. Following consultation with the pulmonologist and infection disease department, the patient was diagnosed with primary tonsillar tuberculosis (with no pulmonary tuberculosis). The treatment protocol was arranged, and the patient received anti-tuberculous treatment for six months, followed up by clinical and laboratory checks to observe their improvement.

## Discussion

The upper aerodigestive tract constitutes the first line of defense against almost all inhalational and related antigenic agents and is exposed to several neoplastic and non-neoplastic destructive lesions, including tuberculosis [8,9]. It is relatively rare for tuberculosis to affect this anatomical area and may be seen in only up to 2% of patients with pulmonary tuberculosis [8]. Isolated tonsil involvement with tuberculosis (primary tuberculosis) is not commonly seen in clinical practice; it accounts for less than 0.5% of all diagnosed cases of tuberculosis cases and is, therefore, a diagnostic challenge [10].

A patient’s medical history alongside a clinical examination may raise clinicians’ suspicion of tuberculosis. The tuberculin skin test and the Interferon-Gamma Release Assay (IGRA) provide evidence for confirmation of the infection; however, both methods are based on cell-mediated immunity [11]. Thus, their accuracy measures are often confounded by the Bacillus Calmette-Guérin vaccination and non-tuberculous mycobacterial infections [12]. Researchers attempted to overcome these limitations, and IGRAs utilizing the region of difference of M. tuberculosis-specific antigens were developed and deemed to be more precise than tuberculin skin tests [13]. However, data remains limited regarding both gold standard tests in the early detection of latent tuberculosis.

In the case of tonsillar tissue, post-operative histopathological examination is a highly useful tool for the detection of unusual inflammatory and/or neoplastic tonsillar lesions [14]. Tonsillar tuberculosis investigations include histopathology, Ziehl-Neelsen staining acid-fast bacillus (AFB), and mycobacterial culture. Other investigations are performed to differentiate the primary lesions from secondary lesions, including chest x-rays, sputum smears for AFB to assess lung involvement, and HIV testing, given that an immunocompromised state is a predisposing factor for tonsillar tuberculosis [15].

The differential diagnosis for tonsillar lesions includes aphthous ulcers, traumatic ulcers, hematological disorders (e.g., leukemia and lymphoma), malignancy, midline granuloma, syphilis, actinomycosis, Wegener's granulomatosis, and Plaut-Vincent's tonsillitis [16,17].

In our case, we excluded all other mimickers by clinical and/or pathological examination where the histopathology revealed a caseating granulomatous reaction, and special staining for AFB on the prepared tissue sections was positive. The two histological features that the pathologist should be aware of and that are highly useful in the diagnosis of tonsillar or lymphoid tuberculosis are variations in the size of granulomas and the presence of caseation necrosis.

Comparing the diagnostic methods, cultures emerge as the gold standard tool, exhibiting the highest sensitivity and specificity. However, according to several studies, the GeneXpert MTB/RIF and MPT64 antigen tests demonstrate similar sensitivities and specificity [18]. The advantage of the GeneXpert MTB/RIF lies in its rapid results, which should expedite treatment initiation.

The medical management of tuberculous tonsillitis includes rifampicin, pyrazinamide, isoniazid, and ethambutol for two months, followed by rifampicin and isoniazid for four months [19]. Our case patient underwent adenotonsillectomy followed by a histopathology examination due to unusual presentation of the tonsils and accidentally discovered tuberculosis, after which they received the treatment protocol and markedly improved.

## Conclusions

Extrapulmonary tuberculosis including the rare entity of tonsillar primary involvement often poses a diagnostic challenge. This article confirms the value of histological examination in detection of tuberculous tonsillitis that was confirmed by AFB staining and the patient received proper antituberculosis treatment in Saudi Arabia with marked improvement. Selective histopathological examination of the tonsillectomy specimens is must for the diagnosis of tuberculous tonsillitis.
